# Quantitative real-time RT-PCR of CD24 mRNA in the detection of prostate cancer

**DOI:** 10.1186/1471-2490-6-7

**Published:** 2006-03-15

**Authors:** M Schostak, H Krause, K Miller, M Schrader, S Weikert, F Christoph, C Kempkensteffen, J Kollermann

**Affiliations:** 1 Department of Urology, Charité – Universitätsmedizin Berlin, Campus Benjamin Franklin, Germany; 2 Department of Urology, Klinikum Fulda, Germany

## Abstract

**Background:**

Gene expression profiling has recently shown that the mRNA for CD24 is overexpressed in prostate carcinomas (Pca) compared to benign or normal prostate epithelial tissues. Immunohistochemical studies have reported the usefulness of anti-CD24 for detecting prostate cancer over the full range of prostate specimens encountered in surgical pathology, e.g. needle biopsies, transurethral resection of prostate chips, or prostatectomies. It is a small mucin-like cell surface protein and thus promises to become at least a standard adjunctive stain for atypical prostate biopsies. We tested the usefulness of real-time RT-PCR for specific and sensitive detection of CD24 transcripts as a supplementary measure for discriminating between malignant and benign lesions in prostatic tissues.

**Methods:**

Total RNA was isolated from snap-frozen chips in 55 cases of benign prostatic hyperplasia (BPH) and from frozen sections in 59 prostatectomy cases. The latter contain at least 50% malignant epithelia. Relative quantification of CD24 transcripts was performed on the LightCycler instrument using hybridization probes for detection and porphobilinogen deaminase transcripts (PBGD) for normalization.

**Results:**

Normalized CD24 transcript levels showed an average 2.69-fold increase in 59 Pca-cases (mean 0.21) when compared to 55 cases of BPH (mean 0.08). This difference was highly significant (p < 0.0001). The method has a moderate specificity (47.3%) but a high sensitivity (86.4%) if the cutoff is set at 0.0498. CD24 expression levels among Pca cases were not statistically associated with the tumor and lymph-node stage, the grading (WHO), the surgical margins, or the Gleason score.

**Conclusion:**

The present study demonstrates the feasibility of quantitative CD24 RNA transcript detection in prostatic tissues even without previous laser microdissection.

## Background

Since 2003, prostate cancer has been the tumor with the highest incidence among men in the Federal Republic of Germany. Approximately 40,600 new cases and 11,100 deaths from this disease were reported for this year in Germany [1]. A precise pathological classification is of essential importance for treating both localized and advanced tumors. Established conventional prognostic markers are prostate-specific antigen (PSA) [2], the tumor stage and grade [3], the patient's age [4], and the presence of residual tumor(s) [5].

PSA shows high sensitivity but very low specificity as a serum marker for prostate cancer. This leads to an enormous increase of basically superfluous prostate biopsies and resultant high costs [2].

Although most of the initially diagnosed tumors are organ-confined (pT2 stage), they are still highly variable in their clinical outcome. This points to the shortcomings/limits of the classical prognostic markers and underscores the need for improved markers.

A comparison of benign, organ-confined, and highly advanced tumors using gene expression profiling techniques has recently disclosed several new molecular markers with prognostic value for prostate cancer [6-8]. A similar approach was used to unmask CD24 as a significant predictor of PSA relapse and poor prognosis in low-grade prostate cancer [9]. CD 24 is a small, heavily glycosylated, mucin-like cell surface protein [10]. It is expressed in granulocytes and various developing cells, including pre-B cells, keratinocytes, and renal tubular epithelium, but also in a large variety of malignancies [11-20]. CD24 is a ligand of P-selectin and could thus promote the dissemination of CD24-positive tumor cells [21].

We evaluated the expression of CD24 by quantitative fluorescence-based real-time RT-PCR (LightCycler) as an alternative to the predominant immunohistolological staining procedures (CD24-specific monoclonal antibodies).

Our aim was to determine whether QRT-PCR-based detection of CD24 will yield data with sensitivity and specificity comparable to those in existing reports based entirely on immunohistochemical staining. In this case, quantitative CD24 mRNA evaluation could supplement the pathologist's subjective analysis in doubtful cases.

The study thus examined 55 probes obtained in cases of benign prostatic hyperplasia (BPH) and 59 from patients with prostatic adenocarcinoma (Pca).

## Methods

### Patients and tissues

Probe retrieval was done in agreement with the Helsinki Declaration. Institutional review board approval was obtained for this study. All patients signed a consent form approved by the Committee on Human Rights in Research at our institution.

The study examined tissues obtained by transurethral resection (TUR-P) in 55 patients with benign prostatic hyperplasia (BPH) and 59 with prostatic adenocarcinoma (Pca) as well as specimens from radical prostatectomies in 51 cases, and tissue chips from 8 palliative TUR-Ps.

In all cases, tissues were removed and immediately snap frozen in liquid nitrogen and stored at -80°C until further processing. Ten 30 μm frozen sections were prepared from each tissue sample with the aid of a Cryostat 2800 (Leica Instruments GmbH, Nussloch, Deutschland) and stained with hematoxylin-eosin.

These sections were stored for later RNA extraction. An additional 10 μm section was used to analyze tumor components

Samples from cancer patients were microscopically examined by an uropathologist (J.K.).

The examined blocks were divided into two groups based on the percentage of malignant cells they contained:

1: 0 – 50 % malignant cells

2: more than 50% malignant cells

Only specimens with a carcinoma component of at least 50% were selected for further mRNA analysis.

### Follow-up

At the time of sample processing, information on the current PSA and any clinical progression was obtained by telephone. Here a PSA of over 0.02 ng/ml was assessed as progression in the 51 radical prostatectomy cases, whereas progression had to be clinical, e.g. new metastases, to be evaluated as such in the 8 palliative TUR-P cases.

### RNA extraction and reverse transcriptase-polymerase chain reaction (RT-PCR)

RNA was prepared from tissue samples using EZ1 BioRobot™ (QIAGEN, Hilden, Germany) according to the manufacturer's instructions. The RNA concentration and quality (28S/18S) were determined after capillary electrophoresis using RNA Nano LabChip^® ^technology using an AGILENT Bioanalyzer 2100 instrument (Agilent GmbH, Waldbronn, Germany).

### Quantitative RT-PCR

Real-time one-step RT-PCR for detecting CD24 mRNA was performed using a LightCycler thermal cycler system according to the manufacturer's instructions (Roche Diagnostics GmbH, Mannheim, Germany).

Amplification primers for CD24 were 5'-TGAAGAACATGTGAGAGGTTTGAC -3' (sense) and 5'-GAAAACTGAATCTCCATTCCACAA -3' (antisense). A hybridization probe-type protocol (GGATGTTGCCTCTCCTTCATCTTGTACATG-FL, FL probe; 5'-AACTCCAGC-AGATTTAATATTGGCATTCATCA-PH-3', LC Red640-probe) was used to detect the 208 bp product.

For one-step RT-PCR, 250 ng of RNA were first reverse-transcribed into cDNA for 10 min at 55° and later amplified for 45 cycles (1 sec @ 95°C, 15 sec @ 58, 20 sec @ 72°C)

The housekeeping gene was amplified using the porphobilinogen deaminase (PBGD) housekeeping gene kit (Roche Diagnostics GmbH, Mannheim, Germany). To amplify the target gene CD24, the LightCycler RNA amplification kit was used in combination with CD24-specific primers (Roche Diagnostics GmbH, Mannheim, Germany).

### Evaluation

LightCycler software version 3.3 (Roche Diagnostics) was used to analyze PCR kinetics and calculate quantitative data. A standard curve generated in a separate run was loaded into runs of patient samples (without standard curves). Each run included one sample of known concentration and in the range covered by the standard curve, thus allowing estimation of exact copy numbers by the second derivative maximum method.

For each sample, copy numbers of CD24 mRNA were divided by those of PBGD mRNA to normalize for CD24 mRNA expression and thus avoid sample-to-sample differences in RNA quantity.

### Statistical analysis

Data were recorded and statistically analyzed using Microsoft^® ^Excel XP^® ^and SPSS for Windows^® ^in Version 13.0.1. Normal distribution was checked. Statistical differences were detected by Student's t test. Results with a two-sided p value of ≤ 0.05 were assessed as significant. A Wilcoxon test was carried out in cases where a normal distribution was not found. The values are plotted on a ROC curve to analyze the extent to which the relative CD24-mRNA expression can differentiate cancer from benign findings. An area under the curve is calculated (from 0.0 to 1.0), and a coordinate table is used to select a favorable intersection point of sensitivity (ordinate) and specificity (abscissa). This is the cutoff.

## Results

This study included 120 prostatic tissues, of which 114 met our RNA quality standards with regard to the presence of 28S/18S RNA (BPH = 55 of 59; Pca 59 of 61). The median PSA was 2.8 ng/ml in the BPH patients and 9.76 ng/ml in the Pca patients. Table 1 shows the pathological parameters of the Pca patients.

**Figure 1:**
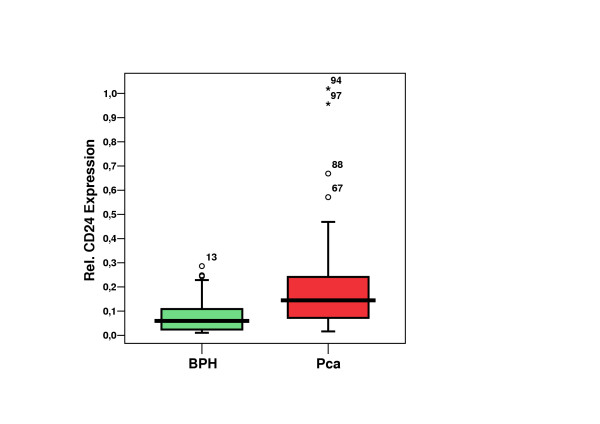
CD24 expression in prostate carcinoma (Pca) cases vs. benign prostatic hyperplasia (BPH) cases. (n = 114; 55 BPH + 59 Pca) Median, Quartiles (25%, 75%), Outliers (5%, 95%), Extremes.

**Figure 2:**
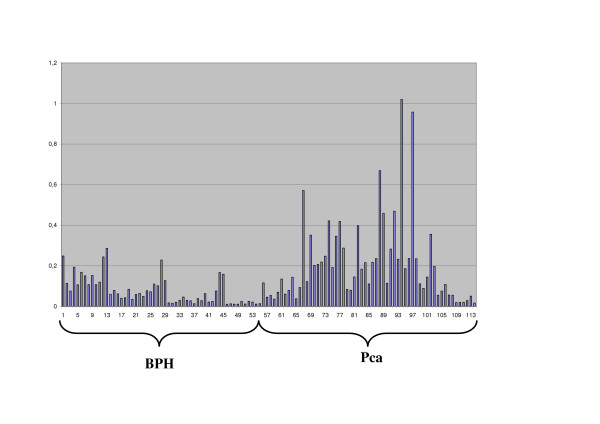
Detailed CD24-mRNA-expression in each case (n = 114; 55 benign prostatic hyperplasia (BPH) + 59 prostate carcinoma (Pca).

Graphs depict relative CD24 expression results in Figures 1 and 2.

**Figure 3:**
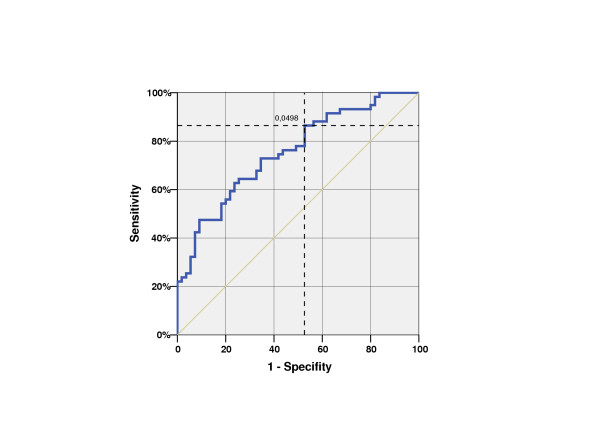
ROC-Curve of CD24 expression (blue); Area under the curve 0,754 n = 114; Positiv (Prostate carcinoma, Pca) 59, negative (benign prostatic hyperplasia, BPH) 55 patients With a cutoff of 0.0498, the relative CD24-mRNA expression had a sensitivity (detection of malignant samples) of 86.4% and a specificity (detection of benign samples) of 47.3%.

**Figure 4:**
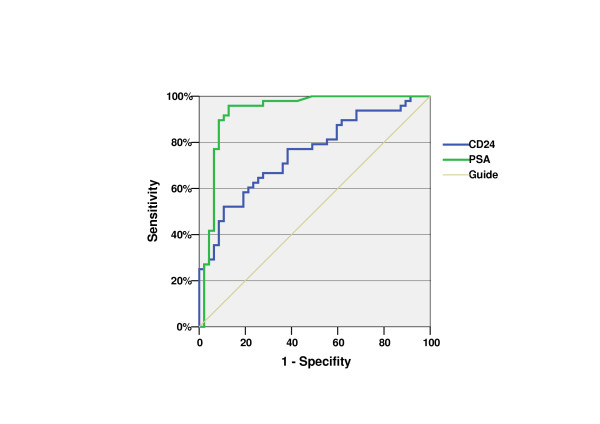
ROC-Curve of CD24 expression (blue) and initial PSA (green); Area under the curve 0.754 resp. 0.932.

The mean relative CD24 expression value was 0.21 (range 0.013–1.02; median 0.14) in the Pca group and 0.08 (range 0.01–0.29; median 0.06) in the BPH group. This Pca patient population had a 2.69 times higher mean CD24 expression for Pca than for BPH. This difference was highly significant (p < 0.0001). Analysis of the CD24-ROC curve (Figure 3) shows a moderate specificity (47.3%) but high sensitivity (86.4%) if the cutoff is set at 0.0498. The Area under the curve was 0.754. The positive predictive value was 64.9% (50/77 patients), whereas the negative predictive value was 75.7% (28/37 patients). Analysis of the ROC curve of the initial PSA yielded an area under the curve of 0.932 (Figure 4).

The Pca group showed no significant correlations between the relative CD24 expression and various clinical parameters, including age, clinical stage, initial PSA, pathological stage, lymph node metastases, margins, grading (WHO), and Gleason Score (Data not shown).

The follow-up (mean 3 years, range 2.4 – 3.3 years) disclosed biochemical progression in only two patients after radical prostatectomy. The relative CD24 expression was within the mean range of all Pca patients at 0.202 in the first case (pat. 70) and 0.42 in the second one (pat. 74). Two patients with palliative resections showed clinical progression. Their relative CD24 expressions were 0.3552 (pat. 102) and 0.075 (pat. 105). These values did not correspond to a significant correlation.

## Discussion

The discovery of prostate-specific antigens (PSA) in 1970 [22] revolutionized the diagnostics of prostatic adenocarcinoma. The good sensitivity but poor specificity of this serum marker has led, on the one hand, to a prognostically favorable stage shift towards increasingly smaller tumors and, on the other hand, to numerous basically superfluous prostate biopsies without tumor cells or with extremely small foci. This offers a great challenge to the assessing pathologists.

In recent years, a great number of new molecular markers (DNA, RNA, protein) for many tumors, including prostate cancer, have been discovered by microarray technologies [23-28]. The clinical value of these upcoming new markers must now be determined in larger clinical studies.

One of these markers is CD24, which was recently described by Kristiansen et al. based on RNA expression profiling. 102 tissue samples from the prostate and 31 samples from lymph node metastases were examined. The immunohistology used in their study achieved a sensitivity of only 48% in the prostate tissue samples and 68% in the lymph node samples. This involved strong staining in only 12% and 29% of the cases and only a very weak reaction in most of them [9] there was no association with tumor stage or grade [8,9]. However, since no benign control tissue was examined in this study, there was no calculated specificity. Kristiansen recently published the results of an exemplary CD24 RT-PCR in several laser-microdissected prostate tissue samples. Here a positive reaction was likewise achieved in only 38.5% of the cases [8]. The high heterogeneity of CD24 expression even within a tumor of the same individual is apparently a problem not only for the CD24 immunohistochemistry of the prostate carcinoma but also for other tumor entities. The general problem of reproducibility of an immunohistochemistry is particularly accentuated by this [9].

Based on the RT-PCR protocol using LightCycler technology, the present study revealed a highly significant elevation of tissue CD24 mRNA expression in Pca patients compared to patients with a benign histology. The sensitivity (correctly detected cancer patients) found in our series (86.4%) is much better than that reported in other series (38.5% and 48% [8,9]). The minimal tumor component yielding a positive result is still unclear. If CD24 mRNA should be detectable not only in tissue but also in prostatic secretions or even urine, the RT-PCR presented here could serve as an important noninvasive supplement to other procedures (digital rectal examination and serum PSA) within the framework of cancer screening. We are currently performing examinations in this connection. However, the moderate specificity (correctly detected patients without cancer) of the technique presented here currently prevents this test from replacing or even supplementing other pathological procedures.

The histology of all patients was known in advance. All BPH 55 patients had either a low preoperative PSA or a previous biopsy to exclude malignancy. Thus we are dealing with a PSA selection. This is corroborated by the far-above-average AUC of the initial PSA (0.932) depicted in Figure 4. Thus, a combination of the predictive values of PSA and CD24-mRNA is not useful, at least not on the basis of the present data.

Laser microdissection was primarily dispensed with, since we wished to streamline the procedure, and Kristiansen et al. have already described the heterogeneity of CD24 expression. We therefore analyzed tissue previously diagnosed for more than 50% tumor infiltration by an uropathologist (J.K.).

The manufacturer designates PBGD as an ideal reference and housekeeping gene and points out that this statement is based on extensive internal series of experiments. The PBGD expression level remained stable with widely varying experimental setups and was also within the RNA expression range of the target gene. No other reference genes were used.

Other study groups found the following results for other tumor entities: Fogel shows that CD24 was expressed in four of six breast cell lines (66.7% sensitivity) [11]. Droz demonstrated that clear cell renal carcinomas always show a strong reaction (100% sensitivity) [19]. The sensitivity was 84% [29] for ovarian carcinomas and 76% [30] for non-small-cell lung carcinomas. Huang showed that CD24 mRNA is expressed in 66% of hepatocellular carcinomas (HCC) [12]. None of the above-mentioned studies examined malignant compared to benign tissue. Thus only conjectures can be made regarding the specificity of the procedures.

We also found no correlation between CD24 expression and clinical or pathological parameters like the tumor stage or metastatic spread. Even specimens from patients with early recurrences did not differ significantly from those obtained in other stages of the disease. Only two of 13 Pca patients with a relative CD24 expression of over 0.3 are currently in disease progression (as indicated by a PSA rise or clinical progression).

Our findings are entirely at variance with results previously described by Kristiansen et al., which led them to assess CD24 as a strong independent predictor of recurrence not only for Pca but also for lung, breast, and ovarian cancer [9,29-31]. Additional multicenter follow-up studies are necessary to confirm the value of CD24 RT-PCR as a prognostic marker.

## Conclusion

Normalized CD24 transcript levels in 59 Pca cases were increased 2.69-fold on the average when compared to 55 BPH cases. This difference was highly significant (p < 0.0001).

The procedure has a very good sensitivity (86.4%) and moderate specificity (47.3%).

CD24 expression levels in Pca cases were not statistically associated with the tumor or lymph node stage or with grading (WHO), surgical margins, or the Gleason score.

The present study demonstrates the feasibility of quantitative CD24 RNA transcript detection in prostatic tissues even without previous laser microdissection. However, the results do not yet signify a major improvement over the usual standard tissue diagnostics.

If CD24 mRNA should be detectable not only in tissue but also in prostatic secretions or even urine, the RT-PCR presented here could serve as an important noninvasive supplement to other procedures (digital rectal examination and serum PSA) within the framework of cancer screening.

## Competing interests

The author(s) declare that they have no competing interests.

## Authors' contributions

Author 1 and 2 carried out the molecular genetic studies (CD24 mRNA) and drafted the manuscript. Author 3 provided central ideas and contributed to the organization of the study. Authors 4, 5, 6 and 7 did tissue collection and workup. Author 8 did the histological analysis, drafted the manuscript and revised it critically.

## Pre-publication history

The pre-publication history for this paper can be accessed here:



## Figures and Tables

**Table 1:** Pathological Classification (59 Prostate carcinoma cases).

**Grading (WHO)**			**Gleason-Score**		
G1	3	5.1%	Median	6	
G2	31	52.5%	Mean	6	
G3	25	42.4%	Range	3 – 9	
Total	59	100%	N	59	

**Local staging**			**Lymph Nodes**		

localized	25	42.4%	Negative	42	71.2%
local advanced	26	44.1%	Positive	14	23.7%
unknown	8	13.5%	Unknown	3	5.1
Total	59	100%	Total	59	100%

**Margins**			**Distant Metastasis**		

Negative	34	57.6	No distant metastasis	57	96.6%
Positive	11	18.7	Distant metastasis	2	3.4
Unknown	14	23.7			
Total	59	100%	Total	59	100%
